# Diagnosis of Pneumonia Due to Invasive Molds

**DOI:** 10.3390/diagnostics11071226

**Published:** 2021-07-07

**Authors:** Carlo Foppiano Palacios, Anne Spichler Moffarah

**Affiliations:** Department of Medicine, Division of Infectious Diseases, Yale University School of Medicine, New Haven, CT 06519, USA; carlo.foppianopalacios@yale.edu

**Keywords:** invasive mold infections, pneumonia, diagnostics

## Abstract

Pneumonia is the most common presentation of invasive mold infections (IMIs), and is pathogenetically characterized as angioinvasion by hyphae, resulting in tissue infarction and necrosis. *Aspergillus* species are the typical etiologic cause of mold pneumonia, with *A. fumigatus* in most cases, followed by the *Mucorales* species. Typical populations at risk include hematologic cancer patients on chemotherapy, bone marrow and solid organ transplant patients, and patients on immunosuppressive medications. Invasive lung disease due to molds is challenging to definitively diagnose based on clinical features and imaging findings alone, as these methods are nonspecific. Etiologic laboratory testing is limited to insensitive culture techniques, non-specific and not readily available PCR, and tissue biopsies, which are often difficult to obtain and impact on the clinical fragility of patients. Microbiologic/mycologic analysis has limited sensitivity and may not be sufficiently timely to be actionable. Due to the inadequacy of current diagnostics, clinicians should consider a combination of diagnostic modalities to prevent morbidity in patients with mold pneumonia. Diagnosis of IMIs requires improvement, and the availability of noninvasive methods such as fungal biomarkers, microbial cell-free DNA sequencing, and metabolomics-breath testing could represent a new era of timely diagnosis and early treatment of mold pneumonia.

## 1. Introduction

The diagnosis of invasive fungal disease and IMIs is evolving, but remains challenging, and lacks the accuracy in diagnoses of infections caused by common pathogens such as bacteria and viruses. Development of non-invasive diagnostic tests is ongoing, as well as attention toward the current delay in standard diagnostic (culture based) methods, difficulty in performing invasive tests due to patient safety, and the variability and heterogeneity in identifying the sensitivity and specificity of the few available fungi serum biomarkers [1,2,3].

The incidence of invasive fungal infections has continued to increase over the last few decades for cancer patients, as well as severely immunocompromised and other susceptible patients, despite major medical advances and interventions to improve the quality of care. The treatment of many conditions has been transformed by the advent of novel cancer therapies, transplantation medicine, and the ability to target a single receptor, cytokine, or cell type using specific agents such as monoclonal antibodies, fusion proteins, and small molecules, but along with the benefits have come risks for fungal infections, invasive fungal infections (IFI), and IMIs [4,5,6,7,8].

Invasive fungal pathogens include primary mycotic organisms, endemic mycosis, such as *Histoplasma capsulatum*, *Coccidioides immitis*, *Blastomyces dermatitidis*, and *Paracoccidioides brasiliensis*, among others, and secondary or opportunistic mycotic organisms such as *Candida*, *Cryptococcus neoformans*, *Pneumocystis jirovecii*, and invasive molds. Of the invasive molds, *Aspergillus* is the most common, followed by *Mucorales* species and an emerging increase in *Fusarium* spp. [9,10,11]. Primary mycosis infection is not exclusively of immunocompromised hosts, whereas secondary mycosis is more common to cause pneumonia in these populations.

Invasive mold infections can cause significant morbidity and mortality in immunocompromised and critically ill patients; they were originally restricted to neutropenic cancer and hematopoietic stem cell transplants (HSCT) patients, but recently have also affected patients with solid tumors and solid organ transplants (SOT), autoimmune disease, congenital immune disorders, and in ICU settings. Most recently, patients with COVID-19 have been recognized as a risk for invasive mold infections. Pneumonia is the most common manifestation of IMI, but mold infections can affect almost any organ system and, depending on the mold, disseminate infection [1,11,12,13].

Fungi are ubiquitous in the environment, typically found in soil and organic matter, and their conidia can be aerosolized and inhaled into the respiratory system. Exposure of the respiratory system-trachea, bronchi, and lung parenchyma to these organisms is common and many fungal infections present with pulmonary involvement [1,14]. Pneumonia can cause life-threatening infection, and diagnostic approaches are challenging due to non-specific clinical manifestations, limited sensitivity, and concerns about specificity of many of the existing diagnostic methods [8,11,12,13,15].

*Aspergillus fumigatus* and other *Aspergillus* spp. (*A. flavus*, *A. terreus*, and *A. niger*) are the predominant pathogenic molds to cause pulmonary disease. Agents of mucormycosis, such as *Mucor* spp., *Rhizopus* spp., and *Lichtheimia* spp., are responsible for 5–15% of IMI cases, while *Fusarium* spp. and *Scedosporium apiospermum* account for a variable proportion of IMIs, depending on the geographical area [10,11,12,15,16]. A combination of testing strategies is the hallmark for accurate IMI diagnosis and essential skills benefit clinicians dealing with at risk patient populations.

The available diagnostic tests and criteria for convincing diagnosis of IFI and IMI, including a definition of invasive fungal infection, have not kept pace with other advanced technologies and therapeutic options available for the at risk populations [17,18,19,20,21]. There is a need for the improvement of current diagnosis for IFI and IMIs, especially noninvasive methods, as well as a call to action for the development of rapid tests, to hasten decision-making in the therapeutic management of this vulnerable population.

In this review, we will provide an overview of populations at risk for IMIs; clinical manifestations of pneumonia due to mold infections in immunocompromised patients, focusing on diagnostic methods for aspergillosis, but also mucormycosis; and an assessment of imaging findings, indirect fungi biomarkers, and molecular diagnostic tests. In addition, we will recommend a step wise diagnostic approach, focusing especially on helping clinicians when treating patients at risk for these infections.

## 2. Populations at Risk and Clinical Manifestations of Pulmonary Aspergillosis and Pulmonary Mucormycosis

The identification of patients at the highest risk for development of IMIs can be accomplished in a number of ways, including multiple available testing and the identification of host and clinical risk factors through examination of vast amounts of patients data [18].

Risk factors for early and late mold infections include host variables, underlying diseases leading to HSCT, therapeutic modalities, immunosuppressive agents employed during transplantation, and complications related to transplantation, including HSCT and SOT. Variables include high doses of cytotoxic agents, body radiation, corticosteroids, cytopenia (probably the most important determinant for IMI is prolonged neutropenia), type of organ transplanted, factors impacting polymorphonuclear (PMN) cells, monocytes, and cellular antifungal defenses. Recognition of these risk factors for IMIs may enable the development of tailored strategies to prevent and diagnose mold infections in these relevant populations [15,22,23,24,25].

An understanding of the host is an essential first step in clarifying the epidemiology, clinical manifestations, and management of fungal infections [2,26]. Briefly, numerous mechanisms of innate and adaptive immunity serve to protect the healthy host from infection; and antifungal defenses exist in a redundant, overlapping network, with primary protection occurring through mucosal immunity, phagocytic cells of the innate immune system, and via a well-coordinated adaptive cellular immune response.

Epithelial cells that line the airways can contribute to innate immune defense, secreting antimicrobial effectors that have activity against molds. However, some fungi can invade epithelial cells, potentially to obtain protection against host defenses. A subsequent line of defense is provided by professional phagocytic cells lining the airways; and immunologic defenses of macrophages provide both antifungal effector activity and serve to coordinate recruitment of other important innate immune cells such as PMNs and natural killer (NK) T cells by secretion of multiple cytokines and chemokines. All impact the immunologic defenses against fungi and underpin susceptibility or resistance to pneumonia [26].

The vulnerable IMI populations include HSCT recipients, oncology patients, especially hematologic malignancies, and patients receiving immunosuppressive drugs (e.g., anti-tumor necrosis factors and monoclonal antibodies), and SOT [11,12,22]. Most cases of IMIs are caused by *Aspergillus* spp., followed by lower number of cases due to other molds, especially *Mucorales* species [6,12,22].

As the world rolls up its sleeves to combat the COVID-19 pandemic, one challenge is that fungal infections can occur alongside COVID-19, especially in ICU patients and patients with pre-existing co-morbidities. Mold infections have been identified in patients with COVID-19, although a causal link has not been proven. The COVID-19 pandemic has exploded since cases were first reported in China in December 2019 and continues to rage worldwide. An abrupt increase in the number of opportunistic fungal infections and outbreaks has been observed; in particular, several cases of COVID-19 associated pulmonary aspergillosis (CAPA) and COVID-19 associated mucormycosis (CAM) have been described. Multiple factors are associated with or lead to the development of those entities, including glucocorticoids and uncontrolled glucose, viral-induced lymphopenia, and direct damage to the airway epithelium, enabling *Aspergillus*, as well as *Mucorales*, to invade tissue and promote pulmonary aspergillosis or pulmonary mucormycosis (PM) [27,28].

### 2.1. Invasive Pulmonary Aspergillosis

The first human case of aspergillosis was described in 1842 by Bennett in Edinburgh [29], based upon a microscopic examination of sputum from a patient with several aspergillomas in tuberculous cavities. It was not until the introduction of corticosteroids and cytotoxic chemotherapy in the 1950s that the first cases appeared of invasive pulmonary aspergillosis (IPA), the opportunistic infection which we now clinically recognize [30].

*Aspergillus* has emerged as one of the most common causes of infectious death in severely immunocompromised patients, with mortality rates approaching 70% in patients with leukemia and recipients of HSCTs. Approximately 15 to 20% of patients with leukemia die of fungal pneumonia caused by *Aspergillus* spp. Similarly, in allogeneic HSCT recipients, 15 to 30% of deaths are caused by refractory fungal infections, mainly caused by *Aspergillus* spp., and most of these infections occur late in the post engraftment period in the setting of graft-versus-host disease (GvHD) [31,32,33].

Of the nearly 200 *Aspergillus* species, only 20 have been recognized as pathogenic in humans. *Aspergillus fumigatus* is the most frequently identified of these species; however, other species have emerged as important pathogens in immunocompromised individuals, such as *Aspergillus flavus*, *Aspergillus niger*, *Aspergillus terreus*, and *Aspergillus nidulans* [10,32,34].

Invasive pulmonary aspergillosis is the most common manifestation of *Aspergillus* spp. infection in immunosuppressed patients. It typically occurs following inhalation of *Aspergillus* conidia. Once germination of conidia occurs, *Aspergillus* hyphae invade pulmonary arterioles and lung parenchyma, leading to ischemic necrosis. Hematogenous dissemination with thrombosis, hemorrhagic infarction, and invasion of distant organs may result from invasion of the arterioles. Damage to respiratory epithelium due to radiation therapy, chemotherapy, GvHD, or prior infection (e.g., RSV, influenza, and CMV) may facilitate attachment of *Aspergillus* conidia to the respiratory epithelial surface. Thus, once *Aspergillus* has invaded the respiratory tract, control of the infection is highly dependent on functional adaptive immune responses as described [10,31,32]. Iatrogenic suppression of protective Th1 responses is common, particularly in the setting of chronic GvHD (an excessive Th1 response of allograft T lymphocytes) treated with high-dose corticosteroids and/or other immunosuppressive regimens [10,33,35].

Identification of clinical risk factors for IPA has been well studied in HSCT recipients, and important risk factors include prolonged neutropenia, corticosteroid use (dose and duration), underlying diseases and transplant-related variables [2]. The “classic” groups of patients at risk for IPA include those with prolonged, profound neutropenia due to a hematological malignancy (5–25% risk), aplastic anemia recipients of allogeneic HSCTs (5–30% risk), or lung transplants (17–26% risk); those with AIDS, severe combined immunodeficiency, or CGD (25–40% lifetime risk); burn patients; and patients receiving chronic corticosteroids. IPA has been increasingly described in new groups of patients, such as systemic lupus erythematosus, Crohn’s disease, and rheumatoid arthritis who received treatment with TNF inhibitors (e.g., infliximab) [4,6,10,33].

Clinical suspicion is the first step in diagnosis. As these infections have diverse etiologies that depend, in part, on different epidemiological settings, it is important to obtain a careful history, information about the patient’s immune status, and geographic place of residence.

Clinical symptoms and signs associated with IPA are notoriously vague but may begin with fever (unless the patient is receiving corticosteroids), which may be followed by a mild nonproductive cough suggestive of bronchitis. As the disease progresses, additional symptoms appear. Pleuritic chest pain and progression to pneumonia can occur within 1 to 2 days, and slight hemoptysis may suggest pulmonary infarction. Cavitation tends to occur in patients if immunosuppression is decreased, such as in leukemic patients during recovery of bone marrow hematopoiesis, or when steroid-based therapy is reduced significantly. Cough, sputum production, and pleural effusion can sometimes be minimal or absent; approximately 25–33% of patients initially have no symptoms attributable to IPA. Invasive aspergillosis (IA) must be strongly considered when dealing with susceptible patients for whom treatment with broad-spectrum antibiotics fails. Early intervention could be lifesaving [6,10,14,30,33].

### 2.2. Pulmonary Mucormycosis

Pulmonary mucormycosis was first described by Fürbinger in 1876 in Germany, after observing a patient who died of cancer and in whom the right lung showed a hemorrhagic infarct with fungal hyphae and a few sporangia [36,37].

Currently, *Mucorales* species are one of the most common mold pathogens after *Aspergillus*, leading to invasive fungal disease in patients with malignancies or transplantation [10,11]. Until the past two decades, most published cases of mucormycosis had been in diabetic patients, which is the most common underlying risk factor globally; however, there has been a notable increase in the rates of mucormycosis in the growing and vulnerable population of patients with impaired immune defenses, primarily those with hematological malignancies and recipients of HSCT or SOTs [36,38,39]. The epidemiology of mucormycosis is evolving as new immunomodulating agents are used in the treatment of not only cancer, but also autoimmune diseases [36,39]. The previous terminology for infections caused by fungi of the order Mucorales, Zygomycosis, is no longer appropriate due to a recent taxonomic reclassification that abolished Zygomycetes as a class [10,16]. Among the Mucoraceae, *Rhizopus oryzae* (*Rhizopus arrhizus*) causes the vast majority (>70%) of Mucorales infections. Other less frequently encountered species of the Mucoraceae family include *Rhizopus microsporus*, *Lichtheimia* (formerly known as *Absidia* species), *Mucor* species, and *Rhizomucor pusillus*. *Cunninghamella bertholletiae* is an increasingly reported cause of mucormycosis and appears to be a virulent species in humans [10,16].

Although still relatively uncommon, mucormycosis is an important and increasing cause of morbidity and mortality in immunocompromised patients. In the current environment, clinicians should consider possible mucormycosis in patients with signs and symptoms of infection, particularly in those with progressing disease despite antifungal treatment covering most other fungal pathogens [16,40]. Risk factors include prolonged corticosteroid use, antifungal prophylaxis with voriconazole, and iron overload and chelation therapy with deferoxamine [41]. Hematological malignancies and neutropenia are associated mostly with PM, while diabetes mellitus with sinusitis and rhino cerebral disease [36].

The predominance of pulmonary disease in at risk patients may be a result of chemotherapy-related defects in innate pulmonary host defenses associated with neutropenia or chemotherapy-induced mucociliary dysfunction. Prolonged and severe neutropenia is the sole identifying risk factor in around 15% of all reported mucormycosis. Nonetheless, similar to aspergillosis, mucormycosis is increasingly reported as a late (1 to 6 months), or very late (>6 months) infection in nonneutropenic, HSCT recipients post engraftment [10,16], especially in the setting of multiple, overlapping, and cumulative mechanisms of immunosuppression during graft versus host disease (GVHD) treatment. Of concern, a growing proportion of PM cases manifest as breakthrough infections on antifungal prophylaxis or treatment effective against aspergillosis, but not mucormycosis (i.e., voriconazole, and echinocandins) [7].

The pathogenesis of PM begins with inhalation of conidia into the respiratory tract, where infection may remain localized in the lung or spreads to the contralateral lung and disseminate hematogenously or via lymphatic spreads. In healthy individuals, mononuclear and polymorphonuclear phagocytes efficiently eliminate fungal spores and hyphae by oxidative and nonoxidative killing mechanisms. Although sporangiospores are the typical infective forms of zygomycetes, angioinvasive hyphal forms are responsible for tissue invasion and dissemination. Spore germination and unopposed hyphal proliferation occur primarily in immunocompromised patients, in whom migration of macrophages and neutrophils, phagocytosis, and killing of spores and hyphal elements, are impaired [42,43]. Quantitative (i.e., neutropenia) or qualitative (i.e., associated with glucocorticoids, hyperglycemia, or acidosis) defects in phagocytic cell activity permit unrestricted growth of the hyphal form and invasive infection. A hallmark of mucormycosis is extensive angioinvasion with resultant vessel thrombosis and tissue necrosis [16,39,42,43].

The clinical manifestations of PM are indistinguishable from IPA [16,43]. Timely diagnosis of PM is challenging, as its symptoms are subtle and nonspecific, even in late stages of infection, especially in patients given anti-inflammatory agents (e.g., systemic corticosteroids or infliximab). The overall mortality rate in patients with PM is high (76%), and it is even higher in severely immunosuppressed patients [40].

Similar to IPA, PM presents with non-specific symptoms such as fever (unresponsive to broad-spectrum antibiotics); a cough, that is typically nonproductive; and severe or subtle pleuritic chest pain. The absence of fever, however, does not rule out mucormycosis, especially in patients receiving corticosteroids. Pulmonary mucormycosis may invade lung-adjacent organs, such as the mediastinum, pericardium, and chest wall. Angioinvasion results in necrosis of tissue parenchyma, which may lead ultimately to cavitation or hemoptysis. Fatal hemoptysis due to fungal invasion of a major blood vessel has been reported occasionally. In patients who have hematologic malignancies, clues for distinguishing PM from IPA clinically are the presence of concomitant pansinusitis, and a history of antifungal prophylaxis with *Aspergillus*-active agents such as voriconazole, Posaconazole, or isavuconazole as well as echinocandins [10,39,40].

## 3. Diagnostic Studies for Invasive Mold Pneumonia

When respiratory symptoms or radiological infiltrates are present in immunosuppressed patients, clinicians must keep in mind that seemingly localized processes can rapidly become diffuse or disseminate in immunologically impaired persons. Thus, a localized pneumonia in this population represents only one point in time, and it must also be approached with a sense of urgency [44].

Early intervention in high-risk patients may be lifesaving, thus an ideal diagnostic test for invasive mold would have high sensitivity and sufficient specificity for early disease. Such a diagnostic would allow a reduction in the use of antifungal agents and be timely and clinically convincing. In addition, the test could be used to follow response to therapy, whereas current diagnostic methods have yet to reach this goal. Diagnosis is based on a combination of clinical risks, symptoms and signs, culture, histopathology, and detection of fungal components. These tests must be interpreted in the context of the patient’s individual risk of infection, to obtain a realistic probability of IA, as well as invasive mucormycosis causing clinical syndromes, including pneumonia [10,14,18,30].

The gold-standard diagnosis of IMIs requires biopsy of tissue for culture and pathology, which is often not feasible due to clinical state of the patient and risk of bleeding. Fungal biomarkers vary in sensitivity, specificity, and predictive value, and some can also be negative in mucormycosis, although in the bronchoalveolar lavage (BAL) those parameters increase. Other tests, such as blood and tissue PCR, as well as metagenomics, are still only research tools, although may at some point be validated and implemented in clinical practice [36,41,45,46].

Imaging is critical to the diagnostic work-up of patients with suspected IMIs. Clinical imaging in suspected IFI has a role in early detection and helps direct further testing. While the presence of specific lesions may increase the likelihood of IFI, the diagnosis by clinical imaging lacks specificity. Findings such as the halo sign (HS), reverse HS, hypodense sign (HDS), and air crescent sign (ACS) may have better discrimination between mold infections and nonfungal pneumonias [47]. Although clinical signs of IMIs are often absent or non-specific at early stages of the disease, suggestive radiological findings are frequently the first trigger to initiate antifungal therapy. Non-enhanced high-resolution CT scanning (i.e., with a slice thickness of 1 mm) currently represents the imaging procedure of choice for the diagnosis of IMI. Other imaging techniques, such as MRI and positron emission tomography (PET) scanning, may also have a place in the diagnosis of IMI [8].

**Figure 1:**
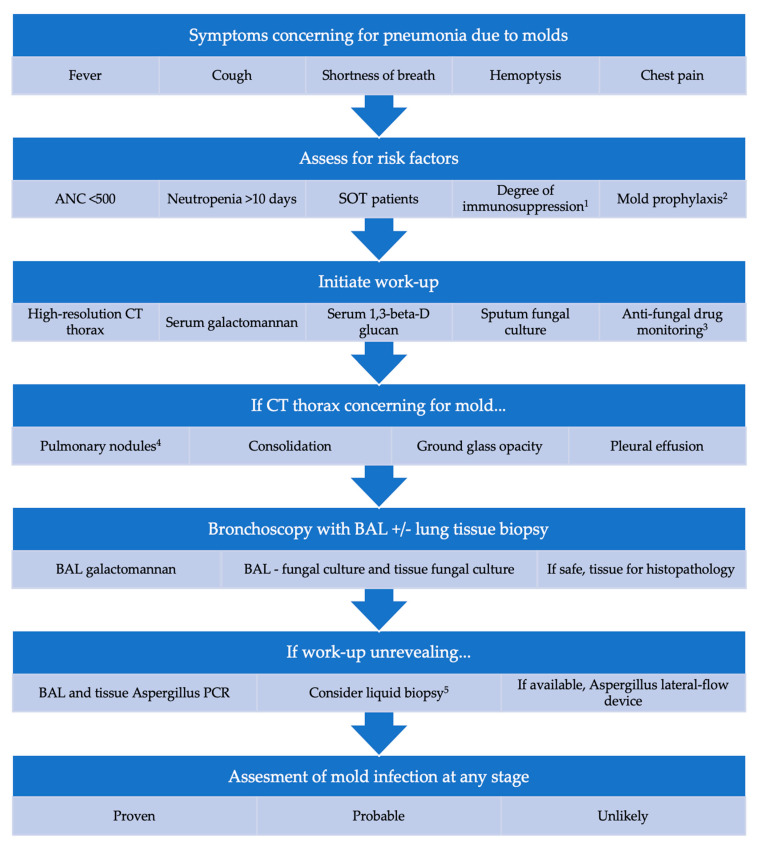
Stepwise diagnosis of pulmonary mold infections in immunocompromised or at-risk host. ^1^ Assess degree of immunosuppression including medications affecting T and B cells, tyrosine-kinase inhibitors, steroids, etc; ^2^ Patient would be at higher risk if not on anti-mold prophylaxis, such as voriconazole, posaconazole, or isavuconazole. Duration of anti-mold prophylaxis is also important; ^3^ Assess anti-fungal drug levels to ensure they are within therapeutic range (plasma drug level monitoring play an increasingly important role in optimizing the safety for voriconazole, and efficacy for posaconazole, and voriconazole of antifungals with significant interpatient pharmacokinetic variability); ^4^ Characteristic description of pulmonary nodules include halo sign, reverse halo sign, air crescent sign, hypodense sign; ^5^ Liquid biopsy or microbial cell-free DNA—sequencing of plasma used for species-level identification of organisms * If ongoing concern for disseminated mold infection, would evaluate for skin lesions on physical exam and rhinosinusitis with CT sinus.

We developed a multifactorial stepwise approach to help clinicians when consulting patients with suspicious of mold invasive pneumonia, including host factors, laboratory, imaging, and medications in use, described in Figure 1.

The development and revision of definitions for IFI will provide homogeneity for research, as well as provide an accurate resource for specialists in the field. Definitions currently in use were developed for research purposes and have been adopted in some clinical practice; however, they should be consider by clinicians in any field or specialties that treat patients with underlying conditions that can lead to IFI as well as IMIs [3,17,48]. Definitions were first published in 2002, revised in 2008, and updated in 2020. There were further refinements to this document, but this new version of definition did not change the classifications of “proven,” “probable,” and “possible” IFD; however, the definition of “probable” has been expanded, and the scope of the category “possible” has been diminished. The category of proven IFD can apply to any patient, regardless of whether the patient is immunocompromised. The probable and possible categories are proposed for immunocompromised patients only [49].

### 3.1. Imaging

The radiographic characteristics of pulmonary mold infections are variable; they lack specificity and are usually related to the type and degree of immunosuppression or underlying host disease.

High resolution CT scanning (with no need for IV contrast due to the inherent high contrast of lung tissue) is considered the optimal imaging for diagnosis of IMIs. It can detect small nodules and typical lesions of IMI such as the halo sign, reverse halo sign, and air crescent sign. These signs are suggestive, but not pathognomonic for IMIs, especially in profound neutropenic patients. CT chest plays a critical role in determining the appropriateness of various downstream diagnostic procedures in patients with IMIs, such as (BAL), percutaneous needle biopsy, and open lung biopsy [10,32,33]. Other imaging methods such as MRI, PET, and PET/CT have disadvantages and sometimes poor resolution for IMIs [8,30,47].

Few studies have investigated the radiologic features of PM, which sometimes cannot be distinguished from IPA. Thus, understanding how to differentiate both could have important therapeutic implications, as voriconazole, the preferred antifungal agent for the treatment of IPA, has no activity in mucormycosis [50,51].

Well-circumscribed lesions (nodules) represent the main radiologic finding of IPA. Nodules may have certain ancillary signs that are more suggestive of IPA, especially in neutropenic patients with leukemia, such as a surrounding halo of ground-glass attenuation, halo sign (HS). The halo sign has been characterized as a discrete nodule of angioinvasive aspergillosis with infarction and coagulative necrosis, surrounded by alveolar hemorrhage. Although this imaging finding is not pathognomonic for IPA, its use of CT for preemptive screening has been shown to improve diagnosis and outcome [51]. It has been documented in only 33 to 60% of patients and is transient. In fact, to be useful for the diagnosis of IPA, CT should be performed within 5 days of the onset of infection, as more than 75% of initial halo signs disappear within a week. Nevertheless, lesions may become bigger in the first 10 days of therapy and with neutrophils engraftment. With neutrophil recovery, these lesions coalesce and cavitate, forming the “air crescent” sign, a classic sign of late filamentous IMIs. This enlargement should not be taken as a sign of treatment failure [10].

Nonspecific and less common findings of IPA include consolidation, cavitary lesions, pleural effusions, ground-glass opacities, tree-in bud-lesions, and atelectasis [47,51]. None of these signs seem to predict the outcome of infection. Radiographic presentation of IA in patients, such as allogeneic stem cell transplant with graft versus host, are less well described and may be more variable. In contradistinction to patients with hematological malignancies, halo signs are rarely observed in SOT patients with IA [14,52].

Few studies have investigated the radiologic features of pulmonary mucormycosis (PM). In one study, the CT findings of PM and IPA were compared in patients with hematologic malignancies, and PM patients were more likely to have multiple nodules (≥10) and pleural effusions [50]. In a second study, there was an increase in the frequency of the reverse halo sign (RHS) in patients with PM compared with patients with IPA (54% versus 6%; *p* < 0.001) [53]. One possible explanation of RHS (an area of ground-glass opacification with peripheral ring of consolidation) in mucormycosis is that *Mucorales* species penetrates the septal wall from the lumen of a capillary or the small vasculature. Thus, a difference in the intensity of penetration and angio-invasiveness between both molds could underlie the difference in the RHS between PM and IPA. Further correlation studies are needed [53].

### 3.2. Microbiological Diagnosis

Diagnosis using culture data for mold pneumonia is limited by poor sensitivity and by the extensive time to result, particularly in light of the prognostic importance of early diagnosis [18]. However, culture remains the gold standard for diagnosis of fungal infection and can be used to determine fungal susceptibility to drugs [8].

Fungal cultures of lower respiratory secretions collected by bronchoscopy and BAL fluid are part of the diagnostic work-up of invasive pulmonary mold infections. The yield of BAL culture is notoriously low, with a sensitivity of 20–50% [8,18,54]. Early performance of CT-guided fiberoptic bronchoscopy with BAL in immunocompromised patients with pulmonary infiltrates has been shown to improve the diagnostic yield of this procedure. Ultimately, open lung biopsy may be required for a definitive diagnosis of IPA, and may not be feasible until late in the course of infection or recovery of pancytopenia [10,32,33].

Respiratory cultures should be carefully interpreted, as it may be difficult to differentiate between fungal colonization and invasive fungal infection [33], particularly in SOT and lung transplant recipients, although the positive predictive value may increases with degree of immunosuppression up to around 77% in patients with hematologic malignancies and HSCT [8,14,55].

In general, for respiratory samples, the most purulent and/or bloody portions of the specimen are inoculated directly onto specialized fungal culture media containing antibiotics to inhibit overgrowth with bacteria. Fungi are difficult to cultivate, and frequently do not grow, despite the use of specialized media and appropriate handling of specimens [1].

Recovery of molds in blood cultures is extremely rare, as most fungi do not usually sporulate in blood, with the exception of *Fusarium* spp. Fungal blood cultures have a lower sensitivity of <10% for *Aspergillus* species [33].

Little improvement has been made over the past few decades in the diagnosis of mucormycosis, which still relies on histopathology and traditional culture methods.

### 3.3. Fungal Biomarkers

In recent years, efforts have been directed toward identifying non-culture-based markers for the rapid and reliable diagnosis of mold infections [10,18]. Diagnostic tests for invasive fungal infections include the galactomannan (GM) enzyme immunoassay, 1,3 β-d-glucan (BDG), a combination of both, and a lateral flow device [3]. Galactomannan, a cell wall component of *Aspergillus* species, is most sensitive for diagnostic of aspergillosis in neutropenic patients with hematologic or oncologic disorders. The role of BDG remains unclear, particularly in organ transplant recipients and other non-neutropenic patients.

For the diagnosis of IA, the performance of GM and BDG testing in serum, as well as GM in BAL, has been evaluated in multiple studies and summarized in several meta-analyses. The major conclusions of some of the meta-analyses showed that, in general, among high-risk patients with hematological malignancies and chemotherapy-induced neutropenia or allogeneic HSCT, the GM and BDG tests have a similar performance, with limited sensitivity (60–80%) and a specificity of 90% [20,21,56]. Insufficient data are available to assess the performance of these tests in solid-organ transplant recipients and other populations of immunocompromised patients. When evaluating meta-analyses that compare diagnostic tests modalities, rather than meta-analyses of randomized controlled trials, heterogeneity of results has been observed depending on the study design (cohort versus case–control studies), the type of patient population (onco-hematological versus other), the method of screening (monitoring or punctual testing), and the criteria used to define a positive test (cut-off of positivity, and number of positive tests) [57].

Although not yet approved, lateral flow devices are a point-of-care immunochromatographic assay to detect an extracellular mannoprotein specific to *Aspergillus* species, and they perform particularly well when combined with GM or PCR for both serum and BAL samples [19,20,21].

#### 3.3.1. Galactomannan

Galactomannan makes up a major part of the cell wall *Aspergillus* spp. and is a polysaccharide consisting of a mannose backbone and a variable number of galactofuran side chains. Galactofuranose-containing polysaccharides vary in size and are secreted by the fungus during growth. It is therefore an attractive biomarker to detect the presence of *Aspergillus* in disease [14,58,59].

A commercially available serum test for GM is a sandwich enzyme-linked immunosorbent assay (ELISA) (Platelia *Aspergillus* EIA), based on the EB-A2 rat monoclonal antibody. The test (serum/plasma and BAL fluid) has been approved as an adjunct for the diagnosis of IA when used in conjunction with other diagnostic procedures. The assay detects a spectrum of galactofuranose-containing molecules and, therefore, is not specific for *Aspergillus*. Several causes of false-positive results include blood transfusions and other blood products, beta-lactam antibiotics, food additives (such as sodium gluconate) [60], and cross-reactivity with other fungi and invasive molds [8,19,20]. False negative results are linked to the pathogenesis of IA, with different degrees of angio-invasion and dissemination according to the level of immunosuppression of the host [8,19,20].

For the diagnosis of IA, serum or plasma GM has an overall sensitivity of 48–92% and specificity of 85–95% [61,62]. The recommended cut-off value for a positive serum GM test is a single value ≥0.7 or two consecutive values ≥0.5; and for BAL fluid is ≥0.8–1.0 [20]. Recent review suggests using a cut-off value of 1.0 to increase specificity [19]. Among patients that are neutropenic, GM had a pooled sensitivity of 61% for patients with probable or proven IPA [62]. The performance of GM in BAL has been assessed in studies and has an overall performance of 85% sensitivity and 90–95% specificity. A higher cut off (OD 1.0 versus 0.5) improved specificity to close to 95% [63,64].

#### 3.3.2. 1,3-β-d-Glucan

1,3-β-d-glucan is a polysaccharide that is a predominant and specific constituent of the cell wall in most fungi. It is thought that fungi release BDG as part of the infection process. BDG can be detected in serum during (IFI), serving as a biomarker for diseases such as IA [20]. Mucorales, and some basidiomycetous yeasts such as *Cryptococcus* spp., are not usually detected by BDG testing as the polysaccharide is not a major cell wall component of these fungal species [20,65].

A colorimetric assay for the detection of BDG, such as Fungitell, does not directly detect BDG; rather, it measures BDG-mediated activation of the coagulation cascade in an amoebocyte lysate of the horseshoe crab. The Fungitell assay is mostly used in the USA and the cut-off recommended by the manufacturers is 80 pg/mL for a positive test and <60 pg/mL for a negative test, with an intermediate “grey zone” between 60 and 80 pg/mL. The sensitivity and specificity rates for this test in limited studies have ranged from 60 to 80%, and from 80 to 90%, respectively, for diagnosis of invasive fungal infections. Among hematologic cancer patients, BDG has a sensitivity of only 61%, but a specificity of up to 99% (especially with two consecutive tests). In SOT patients, sensitivity ranges from 60 to 90%, and a NPV >90% in this population of low incidence of IFI [21]. In IA, the BDG assay has been reported to be less sensitive and reproducible, and becomes positive later during IA disease course, when compared with GM antigen assay for a similar purpose [8,18,21].

It is important to be aware that several factors can induce a false-positive BDG, including blood transfusions and other blood products, IVIG, renal replacement therapy, beta-lactam antibiotics, cellulose-containing dressings, and several Gram-negative infections [60].

### 3.4. Novel Aspergillus Antigens Tests

Novel *Aspergillus* antigen detection tests have been investigated. Recently, IMMY lateral flow assay and the OLM Diagnostics lateral flow device have been approved for use as a diagnostic aid. These are fast and effective alternatives to GM detection and prove to be especially useful for health centers with low sample throughputs. More recently, a lateral flow dipstick assay using a galactofuranose specific monoclonal antibody, which recognizes urine antigens released during *Aspergillus* fumigatus pulmonary infection in animals, demonstrated sensitivity of 80% and specificity of 92%, especially in patients with cancer [66]. However, this assay is not yet commercially available, and is undergoing multicenter validation [19].

### 3.5. Molecular Biology

*Aspergillus*-specific polymerase chain reaction (PCR) testing of blood and BAL fluid has been recently accepted as a mycological criterion for probable IA for research studies [49]. Although the 2016 Infectious Diseases Society of America (IDSA) guidelines on aspergillosis recommends that *Aspergillus* PCR be utilized on an individual basis and in conjunction with other tests and clinical context [6].

The performance of molecular methods for the detection of *Aspergillus* is limited by a lack of standardization in testing methods, difficulty in nucleic acid (NA) with extraction protocols (the fungal cell wall is difficult to disrupt and to penetrate), and contamination rates [1,67].

*Aspergillus* PCR targets a multicopy gene family to enhance sensitivity, and the ribosomal RNA gene cluster (18S/28S rRNA and the internal transcribed spacer (ITS) regions) has been frequently targeted, due to the presence of highly conserved and variable regions. Although *Aspergillus* fumigatus is the most common to cause of IA, it is beneficial to have an assay with additional species of *Aspergillus* which typically cause disease. The use of real-time quantitative (qPCR) can provide rapid species-level identification and generate a quantification cycle (Cq) which is roughly proportional to the fungal burden [67,68,69].

Blood, respiratory, and tissue specimens have been used predominantly for direct detection of *Aspergillus*. In a meta-analysis that combined the results of more than 10,000 serum, plasma, or whole-blood samples from 1618 at-risk patients, the overall sensitivity of a single positive *Aspergillus* PCR was 88%, with a specificity of 75% for proven or probable IA. An important limitation of these studies is the use of conidia as the reference material for assay comparisons, whereas intact conidia are not thought to circulate in the blood of infected patients or to be the predominant form of the organism present in tissue-invasive disease [69,70].

In patients with suspected IA and pneumonia, specimens from the infection site are more advantageous than specimens collected from blood. In a study that compared diagnostic specimen sensitivities in IA, *Aspergillus* PCR from BAL showed better sensitivities than when compared with *Aspergillus* PCR collected from blood (63%) versus (8%), respectively [71]. The optimal use of *Aspergillus* PCR is likely to be in combination with *Aspergillus* antigen detection. In a study which tested BAL, the combination of BAL PCR with GM (Index>1.0) generated 100% sensitivity and 98% specificity [72].

Currently, no guidelines suggest the use of PCR for Mucorales infection [73]. PCR assays have been in development for mucormycosis according to a few selected studies, specifically, experimental studies in mice [74], as well as a retrospective study that evaluated quantitative PCR detection of Mucorales DNA in BAL fluids for the early diagnosis of PM in immunosuppressed patients [75]. In the mouse study, the authors found that the gene family of spore coating encoding proteins (CotH) was feasible as a target for early diagnosis of mucormycosis and, indeed, CotH gene fragments amplified with a high specificity from urine, serum, and BAL fluid samples obtained from mice with mucormycosis. In the human study, a PCR assay that targeted some Mucorales: Rhizomucor, Mucor/Rhizopus, and Lichtheimia, showed good sensitivity and specificity of PCR using the BAL samples, with 24 out of 374 patients with at least one BAL fluid sample having a positive PCR. Mucorales culture was negative for 22 out of 24 BAL fluid samples with Mucorales PCR-positive results. Therefore, Mucorales PCR performed on BAL could provide additional support for diagnosis of mold pneumonia in immunosuppressed patients [75].

### 3.6. Liquid Biopsy/Microbial Cell-Free DNA Sequencing

A challenge of diagnostics is to comprehensively identify pathogens causing infection throughout the body using samples obtained non-invasively. Such approaches have not yet been implemented in immunocompromised patients suspected of IMIs. Sequencing of cell-free DNA (cfDNA) has recently been shown to enable non-invasive diagnosis, including infection due to multiple organisms such as non-*Aspergillus* and *Aspergillus* mold [76,77].

Microbial cell-free DNA sequencing (mcfDNA-Seq) of plasma has been increasingly used as an alternative test for species-level identification of organisms. Fragments of genomic DNA from pathogens causing infection at various locations in the body are found in purified plasma cfDNA, thus raising the possibility of non-invasive detection of a wide range of infections by sequencing the microbial cfDNA [76,77].

A recent retrospective study evaluated the performance of a plasma mcf-DNA-Seq test for diagnosing pulmonary IMI after hematopoietic cell transplant. Results showed sensitivity of 51% among patients with any proven/probable pulmonary IMIs, for detection of ≥1 pathogenic mold. In patients with proven or probable IPA, sensitivity was 31% to detected *Aspergillus* DNA in plasma, while in non-*Aspergillus* pulmonary IMIs, non-*Aspergillus* molds yield a sensitivity of 79%. This study highlights an important tool to implement in patients suspected with IMIs, especially for non-*Aspergillus*. The study suggested that noninvasive mcfDNA-Seq had moderate sensitivity and high specificity, NPV, and PPV for pulmonary IMIs after HCT, particularly for non-*Aspergillus* species [76].

### 3.7. Metabolomics

Breath testing for fungal respiratory infections has great promise as samples can be obtained by noninvasive, repeatable techniques that can be obtained from a wide range of patients, including immunocompromised individuals. Volatile organic compounds (VOCs), products of fungal metabolisms, have an emerging role in the diagnosis of IMIs; although studies are needed to identify and validate VOC from pathogenic fungi.

In an experimental murine model of invasive mucormycosis (IM), Koshy et al. examined breath volatile metabolite profiles, using the three Mucorales species that most commonly cause human disease (*Rhizopus arrhizus var. arrhizus*, *Rhizopus arrhizus var. delemar*, and *R. microspores*), by thermal desorption gas chromatography/tandem mass spectrometry (GC–MS), with mice infected with *Aspergillus fumigatus* used as controls [78]. They also analyzed breath volatile metabolites from five patients eventually diagnosed with proven IM caused by *R. microsporues.* The findings showed that the three Mucorales species had distinct breath profiles of the volatile metabolite sesquiterpene, which could be used to identify these infections in vivo. These profiles distinguished the type of Mucorales infections within the positive breath profiles and from aspergillosis, therefore this method has the potential to diagnose fungal infection non-invasively. Additionally, it could be used to diagnose IMIs in high-risk populations, such as patients with neutropenia due to treatment for hematological malignancies on chemotherapy, or after hematopoietic cell transplantation. This method is appealing and promising, but needs further evaluation [36,78,79].

### 3.8. Pathology

Histopathology continues to be a rapid and cost-effective means of providing a presumptive or definitive diagnosis of an invasive fungal infection. However, the use of fungal silver impregnation stains (Grocott or Gomori methenamine silver (GMS)) cannot alone solve these challenges, and newer diagnostic techniques may be required.

Identifying fungal elements in histology from BAL or lung biopsy provides proof of fungal lung infection. Routine hematoxylin and eosin (H&E) stains may show only the cell wall with no structures inside, or, occasionally, very degenerate hyphae. Stains that can help highlight the fungal wall include GMS and periodic acid-Schiff (PAS) stains, although PAS gives a better visualization of the surrounding tissue compared to GMS. *Aspergillus* spp. are usually described as thin (3 to 12 μm), septate, acute-angle (450) or dichotomous branching hyphae, nonpigmented (hyaline), while Mucorales are described as nonpigmented (hyaline), and pauciseptate ribbonlike hyphae with right-angle branching. For the specific identification of Mucorales in tissues or to detect dual infections by Mucorales genera and other fungi, it is important to use immunohistochemistry, in situ hybridization, or PCR [36,80].

There has been substantial work on the development of an application of PCR to amplify fungal DNA from both formalin fixed paraffin embedded (FFPE) and fresh tissue. Although such tissue-based molecular assays may not provide a cost-effective tool for routine clinical diagnosis and management of fungal infections, in cases where there is histopathological evidence of a fungal infection without confirmation by culture, molecular tools may assist in the identification of the etiological agent. Thus, fungal elements seen in tissue samples by histopathology and identified by PCR, followed by sequencing, should fulfill the definition of a proven fungal infection, even in the absence of culture [81].

## 4. Conclusions

This article provides the current literature and understanding of pulmonary infections with invasive mold, mostly aspergillosis, but also mucormycosis. In summary, we described host factors that lead to development of IMIs and pneumonia, clinical characteristics in detail, imaging findings, and standard diagnostic test modalities, as well as new diagnostic approaches for vulnerable populations that urgently need new diagnostic techniques for invasive mold pneumonia. The review is also directed to help guide clinicians when dealing with immunocompromised patients with pneumonia, and suspicious of fungal infections, which in many situations can be challenging.
